# Supplementary material for: Impact of disease-associated chromatin accessibility QTLs across immune cell types and contexts
Source: Cell Genom. 2025 Nov 10;6(1):101061. doi: 10.1016/j.xgen.2025.101061 (PMC12926193; doi:10.1016/j.xgen.2025.101061)
Supplement: Document S1. Figures S1–S14 [file mmc1.pdf]

Cell Genomics, Volume *6*

Supplemental information

Impact of disease-associated chromatin accessibility QTLs across immune cell types and contexts

Zepeng Mu (牟泽鹏), Haley E. Randolph, Raúl Aguirre-Gamboa, Ellen Ketter, Anne Dumaine, Veronica Locher, Cary Brandolino, Xuanyao Liu, Daniel E. Kaufmann, Luis B. Barreiro, and Yang I. Li

Supplemental Information for:

Impact of disease-associated chromatin accessibility QTLs across immune cell types and contexts

Zepeng Mu(牟泽鹏)[1,2], Haley E. Randolph[1,3], Raúl Aguirre-Gamboa[4], Ellen Ketter[5], Anne Dumaine[4], Veronica Locher[6], Cary Brandolino[4], Xuanyao Liu[1,4,7], Daniel E. Kaufmann[8,9], Luis B. Barreiro[1,4,6,7,10,†], Yang I. Li [1,4,7,10,†]

1. Committee on Genetics, Genomics & Systems Biology, University of Chicago, Chicago, IL, USA
2. Center for Data Sciences, Brigham and Women’s Hospital, Harvard Medical School, Boston, MA, USA
3. Department of Pediatrics, Columbia University Irving Medical Center, New York, NY
4. Section of Genetic Medicine, Department of Medicine, University of Chicago, Chicago, IL, USA
5. Committee on Microbiology, University of Chicago, Chicago, IL, USA
6. Committee on Immunology, University of Chicago, Chicago, IL, USA
7. Department of Human Genetics, Department of Medicine, University of Chicago, Chicago, IL, USA
8. Division of Infectious Diseases, Department of Medicine, University Hospital and University of Lausanne, Lausanne, Switzerland
9. Centre de Recherche du CHUM (CRCHUM) and Département de Médecine, Université de Montréal, Montreal, QC, Canada
10. CZ Biohub Chicago, Chicago, IL, USA

[†] Corresponding authors

Figure S1. Quality control on scATAC-seq data and genotyping, related to Figure 1. 3
Figure S2. Visualization of cell loadings for the 20 topics in UMAP embedding, related to Figure 2. 5
Figure S3. Topic modeling analysis, related to Figure 2. 6
Figure S4. Topic modeling captures technical variations in a subset of topics, related to Figure 2. 8
Figure S5. Benchmarking strategies to derive gene scores from peak scores in topic modelling with topic k17, related to Figure 2 9
Figure S6. CaQTL mapping using RASQUAL and sc-PME model in harmonized data, related to Figure 3. 10
Figure S7. Permutation analysis for sc-PME model, related to Figure 3. 12
Figure S8. Comparison of sc-PME model with QTLtools, related to Figure 3. 13
Figure S9. Sc-PME model captures distal genetic regulation in open chromatin, related to Figure 3. .. 14
Figure S10. Sharing of RASQUAL cPeaks in cell type pairs, related to Figure 3. 15
Figure S11. Colocalization of caQTL with eQTL and disease GWAS, related to Figure 5. 16
Figure S12. Features of colocalized eQTL and caQTLs, related to Figure 5. 18
Figure S13. Pleiotropy in caQTL-GWAS and eQTL-GWAS colocalizations, related to Figure 5. 19
Figure S14. IBD GWAS locus near CARD9 colocalizes with caQTLs in CD4 T cells, related to Figure 5. 20

Supplemental References **21**

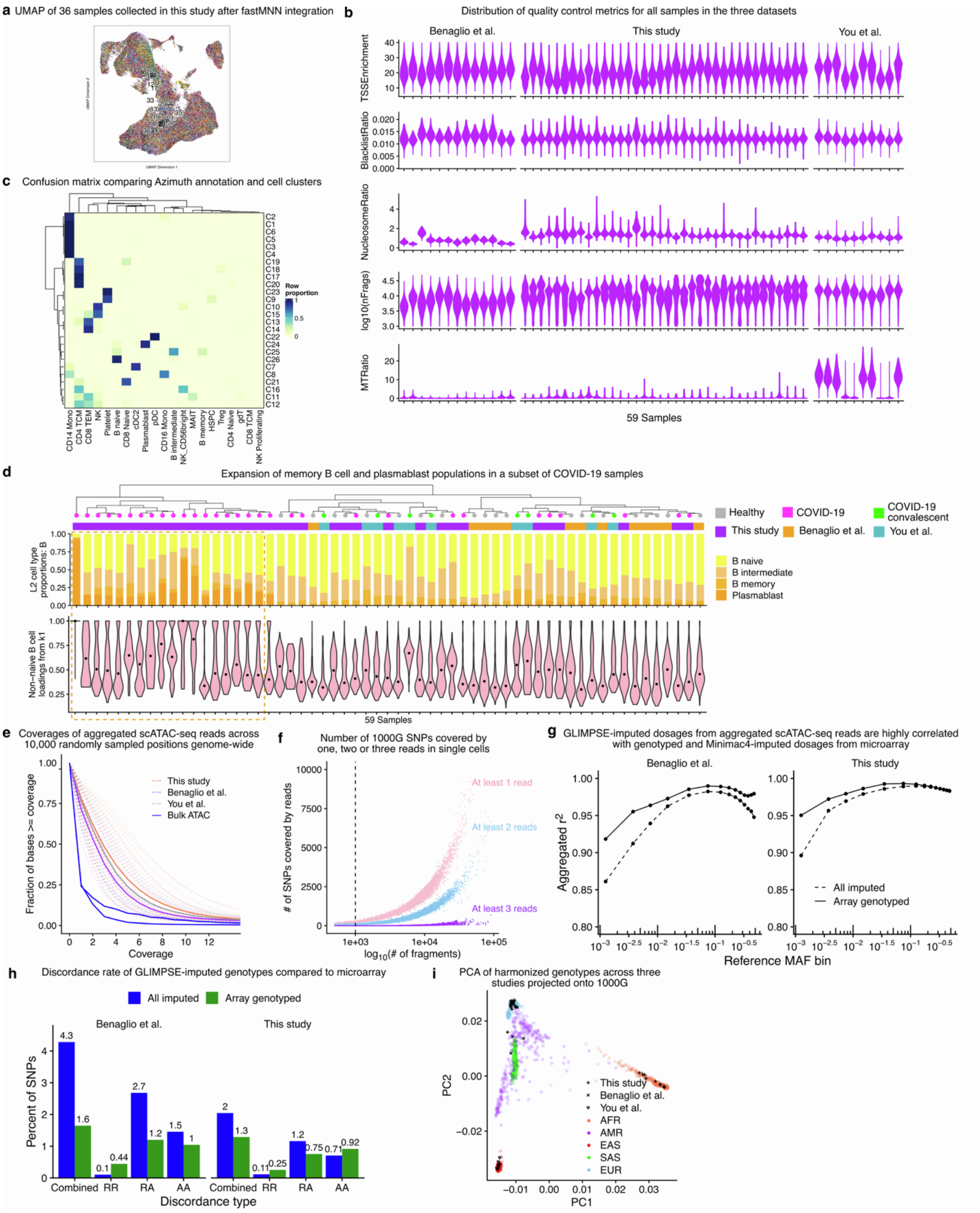

Figure S1. Quality control on scATAC-seq data and genotyping, related to Figure 1. a, UMAP of integrated 36 samples collected in this study. **b**, Violin plots showing the distribution of common quality control metrics for all cells in the three datasets. **c**, Confusion matrix comparing Azimuth L2 annotation and cell clusters. Heatmap is colored by row normalized proportions, such

that the cell-type compositions sum up to one on each row. **d**, Top, bar plots showing expansion of memory B cells and plasmablasts (highlighted in dashed box) in a group of COVID-19 samples. Below, non-naïve B cell loadings calculated from topic k1. Dots on violin plots indicate median value. **e**, Read coverage at 10,000 randomly sampled genome-wide positions in the three scATAC-seq data sets, compared to two public bulk ATAC-seq libraries. Dashed lines show each sample in scATAC-seq data; solid lines show the median of all samples within a data set. **f**, Number of 1000G SNPs covered by at least one, two or three reads in each single cell as a function of the number of unique fragments. Dashed line indicates 1,000 unique fragments, the cutoff we used for filtering low-quality cells. **g**, Mean correlation between GLIMPSE-imputed genotype dosages from aggregated scATAC-seq reads and those genotyped (solid line) or imputed (dashed line) from microarray data in Benaglio et al.[1] and samples collected in this study. **h**, Discordance rate between GLIMPSE-imputed genotype dosages from aggregated scATAC-seq reads and those genotyped or imputed from microarray data in Benaglio et al. and samples collected in this study. **i**, Principal component analysis of imputed genotype from all individuals in this study projected onto 1000G samples.

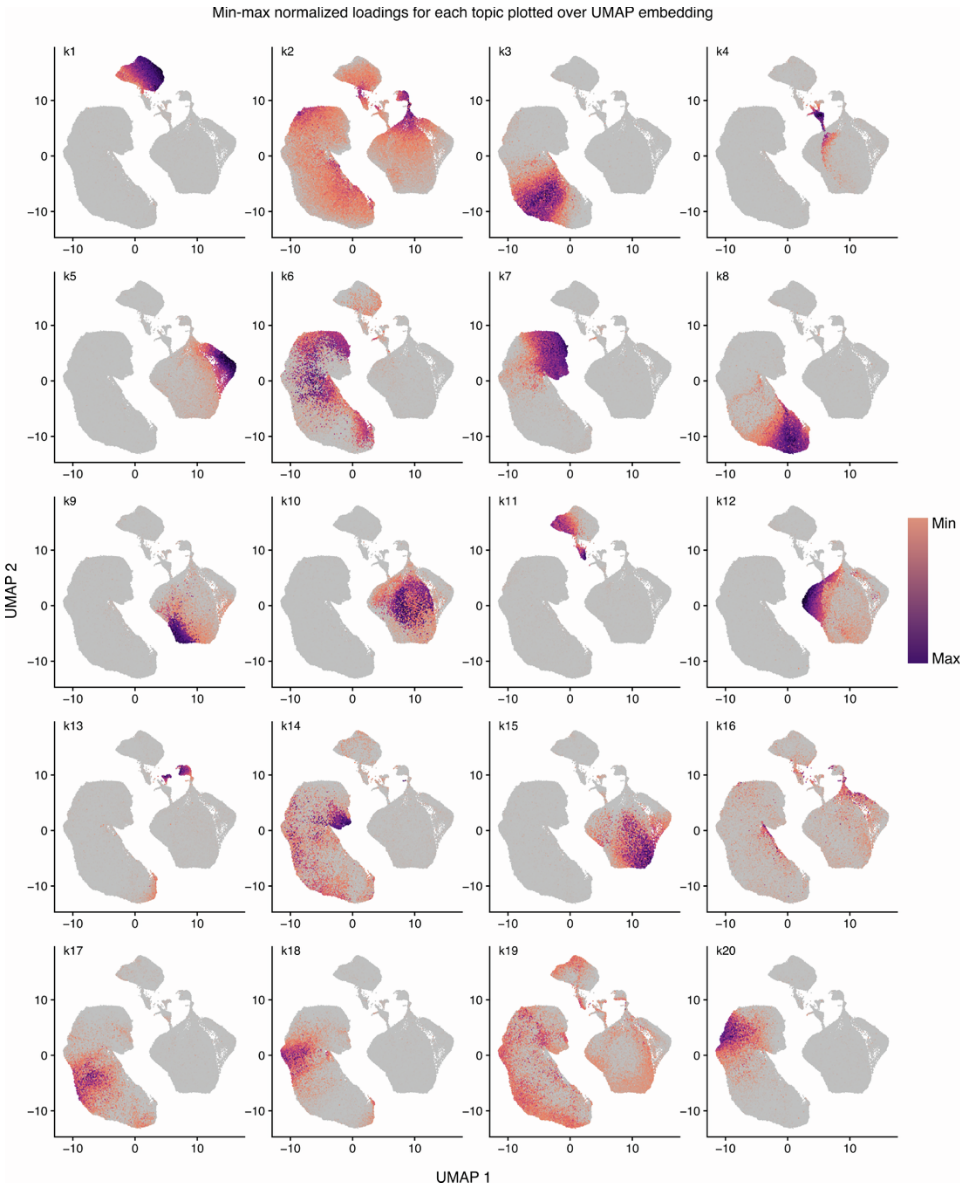

Figure S2. Visualization of cell loadings for the 20 topics in UMAP embedding, related to Figure 2. UMAP plots showing the distribution and quantity of loadings for the 20 topics. Loading scores are min-max normalized for each topic and loading scores below 0.05 in a cell are set to zero for visualization purposes (gray).

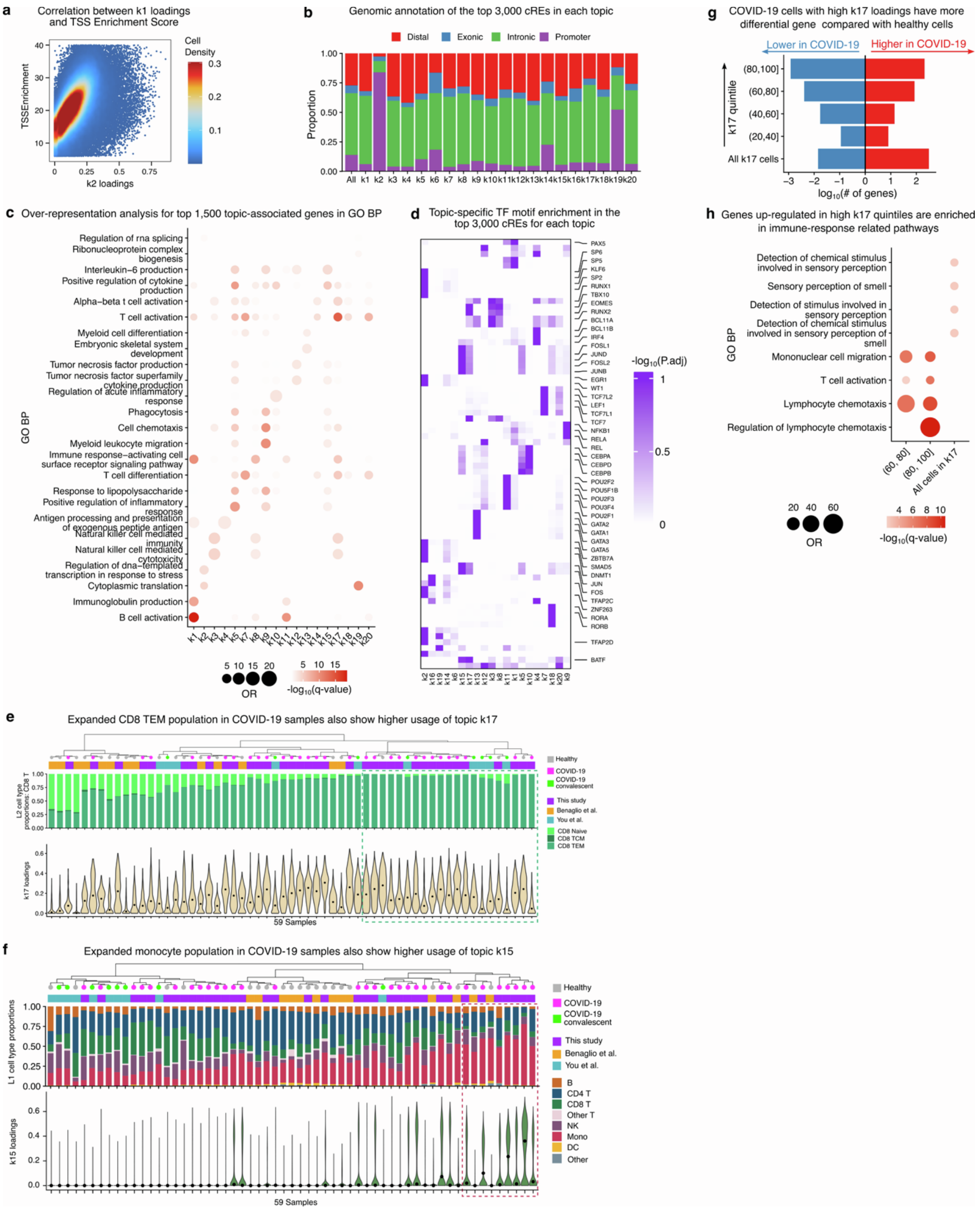

Figure S3. Topic modeling analysis, related to Figure 2. a, Scatter plot showing the correlation between k2 loadings and TSS Enrichment scores. **b**, Bar plot showing the genomic annotation of the top 3,000 peaks in each topic, compared to all peaks, highlighting the over-representation of promoters in k2. **c**, GO Biological Process pathways enriched in top 1,500 scored genes for each topic. **d**, Heatmap of adjusted p-values for TF motif enrichment in top 3,000 peaks in each topic. $-\log_{10}$(P adj.) values are normalized relative to maximum for each TF across all topics. Top five enriched TFs are shown for each topic. **e**, Top, bar plots showing expansion of CD8 TEM

(highlighted in dashed box) in a group of COVID-19 samples. Below, topic k17 loadings. Dots on violin plots indicate median value. **f**, Top, bar plots showing expansion of monocytes (highlighted in dashed box) in a group of COVID-19 samples. Below, topic k15 loadings. Dots on violin plots indicate median value. **g**, The number of differentially active genes in COVID-19 cells in the top 4 k17 quintiles compared to all cells in the first k17 quintiles, plotted together with the number of differentially active genes when all COVID-19 cells in k17 were tested against all healthy cells in k17. **h**, GO Biological Process pathways enriched in up-regulated genes from groups in **g**. Only groups with significantly enriched GO terms were plotted.

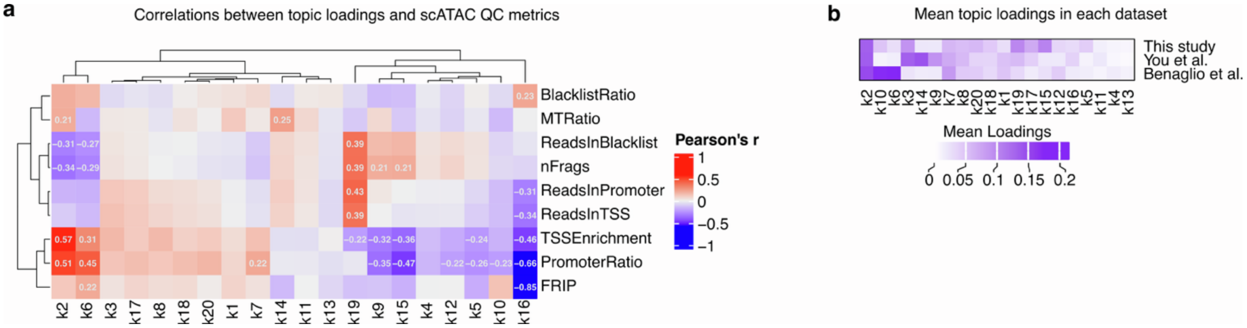

Figure S4. Topic modeling captures technical variations in a subset of topics, related to Figure 2. a, Heatmap for correlation between single-cell quality metric and topic loadings. Pearson's correlation bigger than 0.2 are labeled. **b,** Heatmap for mean topic loadings in each dataset.

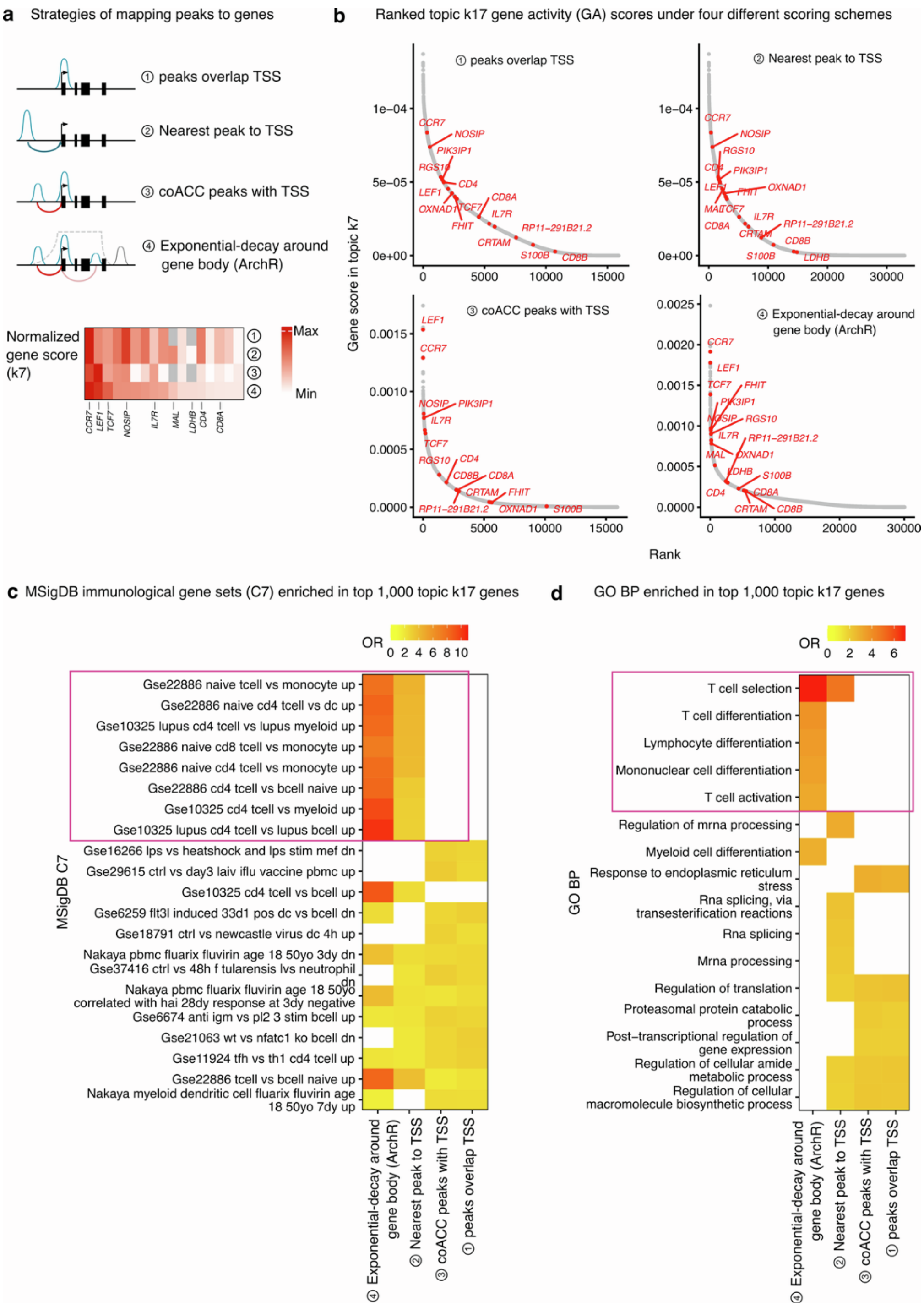

Figure S5. Benchmarking strategies to derive gene scores from peak scores in topic modelling with topic k17, related to Figure 2. a, Top: schematic representations for four strategies used. Bottom: topic k17 is benchmarked against naïve CD4 T cell genes. **b,** Ranked plot for topic k17 genes scores of all genes. Naïve CD4 T cell marker genes are highlighted and labeled in red. **c**, Top five enriched MSigDB immunological gene sets (C7) in each scoring strategy. CD4 T cell relevant gene sets are highlighted in box. **d**, Top five enriched Gene Ontology Biological Process (GO BP) gene set in each scoring strategy. CD4 T cell relevant gene sets are highlighted in box.

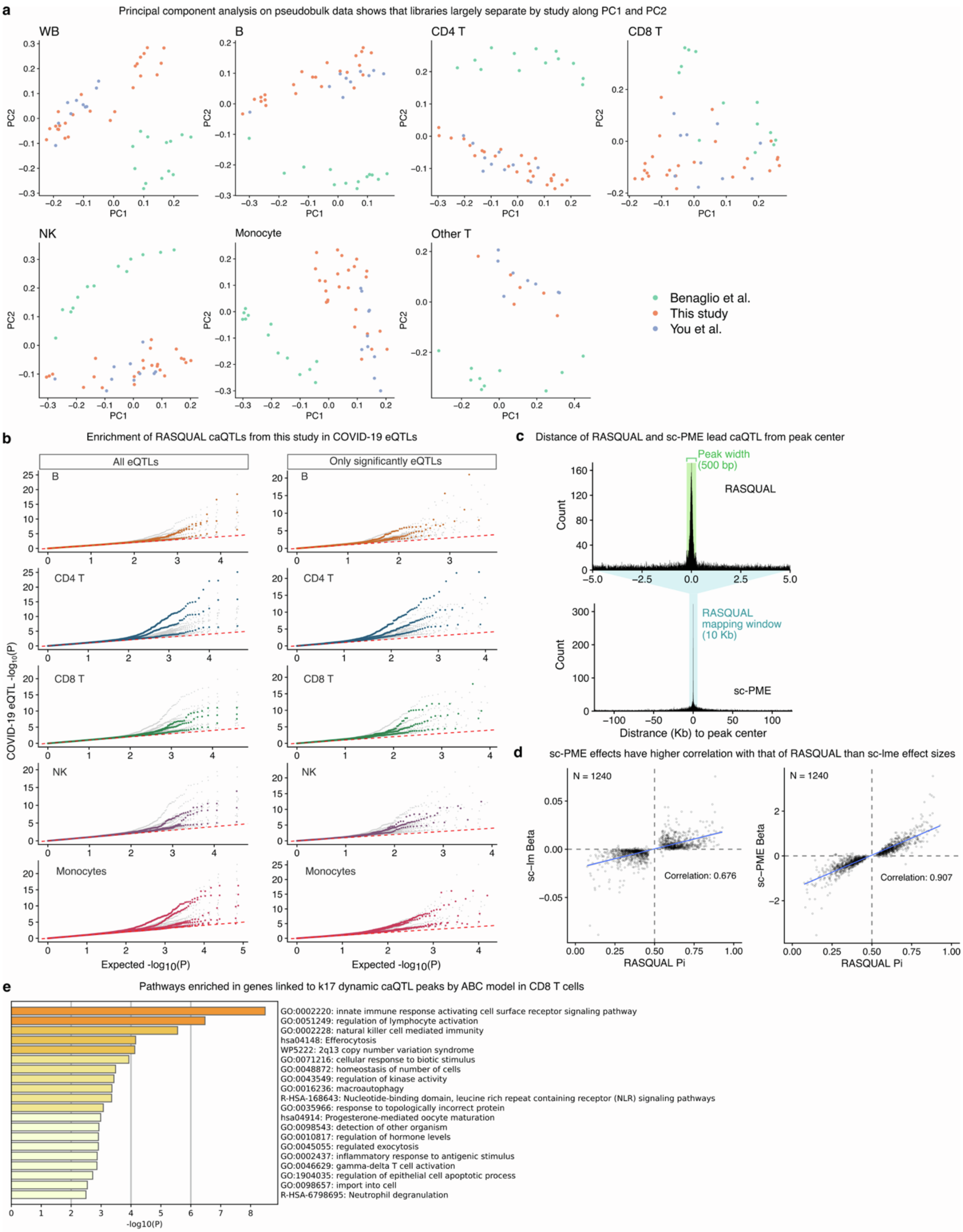

Figure S6. CaQTL mapping using RASQUAL and sc-PME model in harmonized data, related to Figure 3. a, Principal component analysis on pseudobulk count data for aggregated PBMC and six cell-types in which caQTL mapping was conducted. Each sample is colored by study. **b**,

QQ-plot showing the enrichment of caQTLs in our accompanying COVID-19 eQTLs for all eQTLs (left) and conditioning on only significant eQTLs (right). For each cell type in our study, we extracted and plotted eQTLs p-values from all cell types, highlighting matched cell types in colored dots; eQTL p-values from the remaining cell types were colored grey. When all genes are used, caQTLs tend not to have the highest enrichment in eQTLs in corresponding cell types, except for CD4 T and monocyte, due to lower power in the eQTL data (left). We therefore conditioned on only significant eGenes in each cell type to mitigate the differences in power between cell types, and observed larger enrichment of caQTL in eQTLs for matched cell types (right). **c**, Histogram showing distances from peak centers to significant lead caQTL in monocytes from RASQUAL (top) and sc-PME (bottom). Green shaded region highlights peak size (500 bp); blue shaded region highlights RASQUAL mapping window (10 Kb) relative to sc-PME mapping window (250 Kb). **d**, Scatter plots comparing RASQUAL effect sizes (Pi) with sc-lme effect sizes (left) and sc-PME effect sizes (right). Only significant RASQUAL caQTLs on chromosome 1 in monocytes were plotted. **e**, Top pathways enriched in genes linked to peaks with k17-interacting dynamic caQTLs in CD8 T cells.

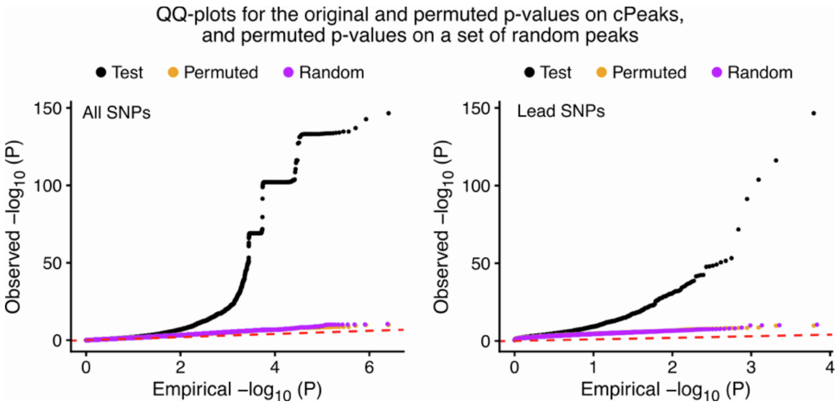

Figure S7. Permutation analysis for sc-PME model, related to Figure 3. Left, QQ-plot for all SNPs in original (Test) sc-PME model and permuted analysis for all cPeaks. Also shown are permutation results for a random set of non-significant peaks (Random). Right, similar to left, showing p-values for lead SNPs only. Significant cPeaks from monocytes on chromosome 1 were used in this analysis.

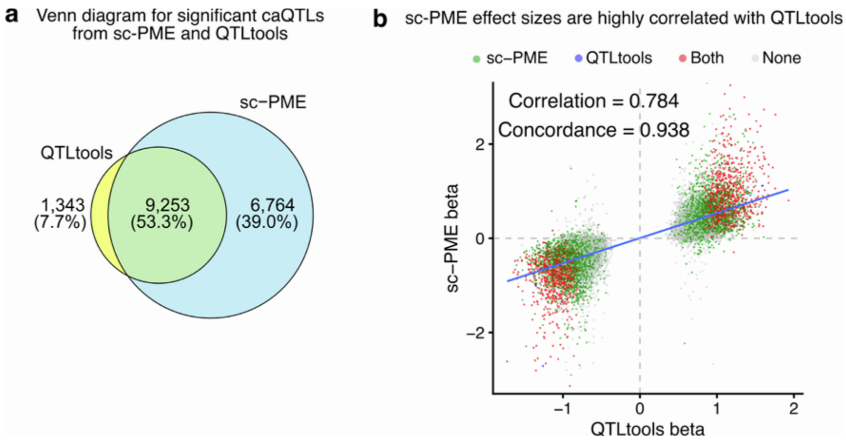

Figure S8. Comparison of sc-PME model with QTLtools, related to Figure 3. a, Venn diagram showing the number and overlap of significant cPeaks found in sc-PME and QTLtools in monocytes. **b**, Correlation in effect sizes from QTLtools compare with those from sc-PME model. Lead SNPs from QTLtools are chosen and plotted.

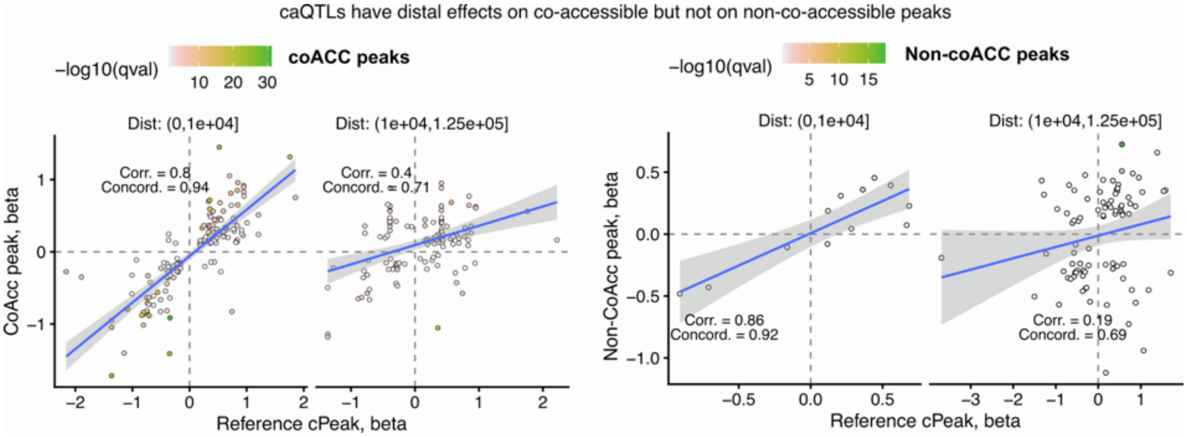

Figure S9. Sc-PME model captures distal genetic regulation in open chromatin, related to Figure 3. Left, correlation between a caQTL effect on a cPeak (reference cPeak) and its effect on a distal, co-accessible (coAcc) peak in monocytes. Pairs of peaks are separated by their distances. 0-10Kb represent the mapping window size in RASQUAL; 10-250 Kb represent that of sc-PME, which cannot be mapped in RASQUAL. Right, similar to left, showing results from a set of non-co-accessible peaks as negative control.

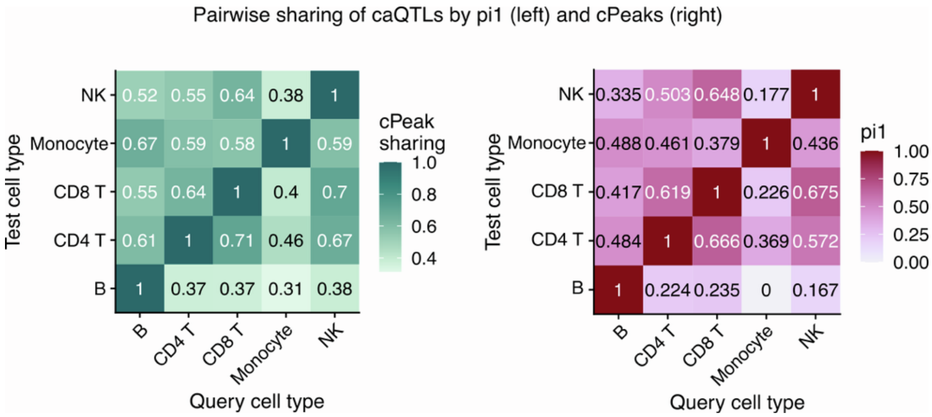

Figure S10. Sharing of RASQUAL cPeaks in cell type pairs, related to Figure 3. Left, pairwise sharing of cPeaks from RASQUAL. Right, pairwise sharing of caQTLs as quantified by pi1 statistics.

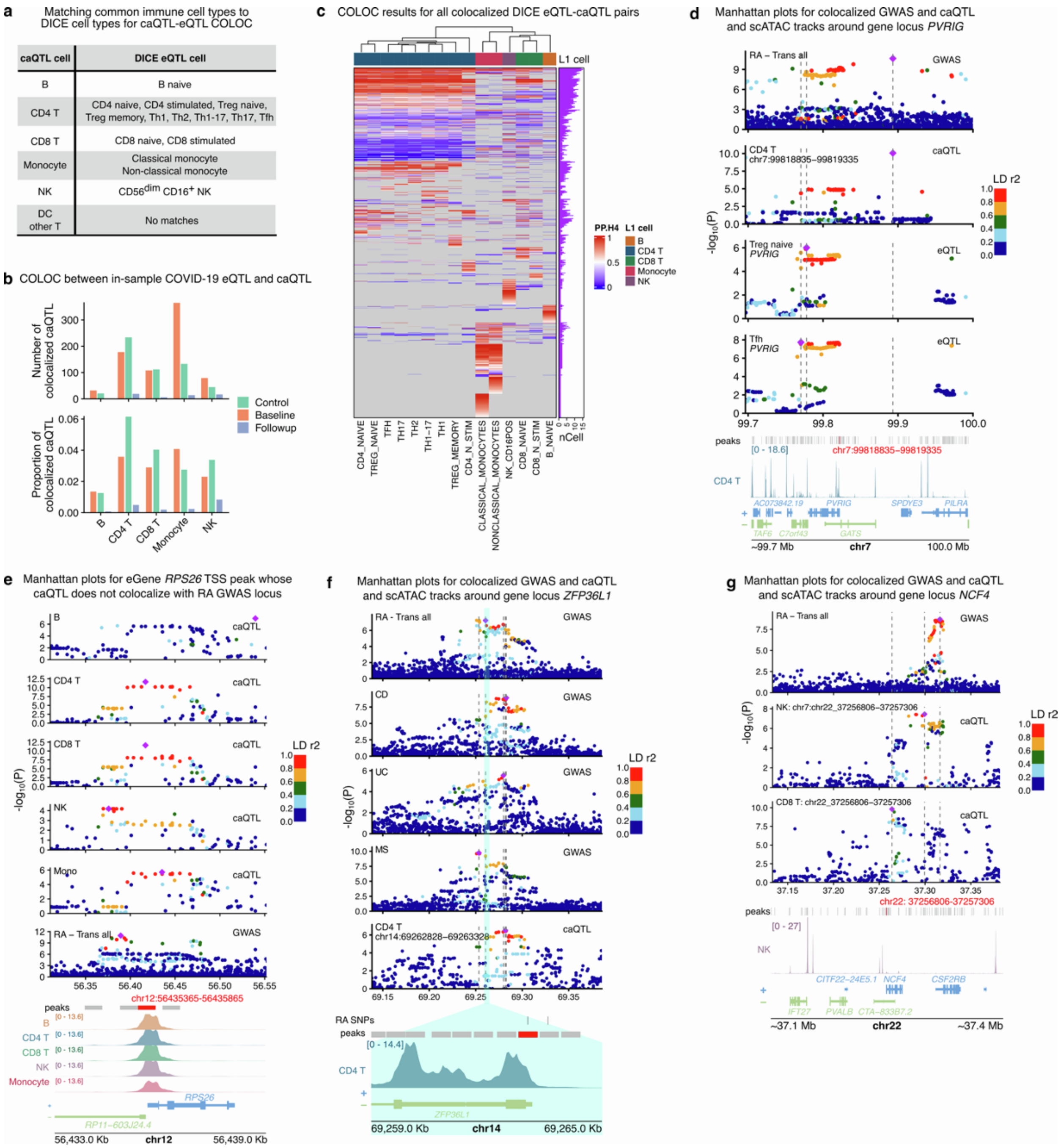

Figure S11. Colocalization of caQTL with eQTL and disease GWAS, related to Figure 5. a, Table summarizing the mapping of 15 immune cell-types in DICE data to the seven common cell-types used in caQTL mapping. **b,** Top, number of caQTLs that colocalize with our in-sample sc-eQTLs in five common immune cell types. Bottom, proportion of tested caQTLs that colocalize with our in-sample sc-eQTLs in five common immune cell types. **c**, Heatmap showing PP4 for all colocalizations between our caQTL and DICE eQTL. caQTL-eQTL pairs not tested in colocalization are colored grey. **d**, Manhattan plots for RA GWAS locus (7:99893148) and colocalized caQTLs (chr7:99818835−99819335) and eQTLs (*PVRIG*), with genome browser tracks for scATAC data in CD4 T cells. **e**, Manhattan plots for an RA GWAS locus near TSS of *RPS26*; the GWAS locus colocalized with *RPS26* eQTLs in DICE. The TSS peak is accessible and has caQTL in all five cell types, but the caQTLs do not colocalize with the RA GWAS. **f**,

Manhattan plots for RA, CD, UC, MS GWAS loci and colocalized cPeak (chr14:69262828−69263328). Genome browser track shows promoter region of *ZFP36L1* near the colocalized peak and scATAC data in CD4 T cells. **g**, Manhattan plots for RA GWAS locus (22:37316259) and colocalized cPeak (chr22:37256806-37257306) in NK cells in the *NCF4* locus. Genome browser track shows scATAC data in NK cells in the same region.

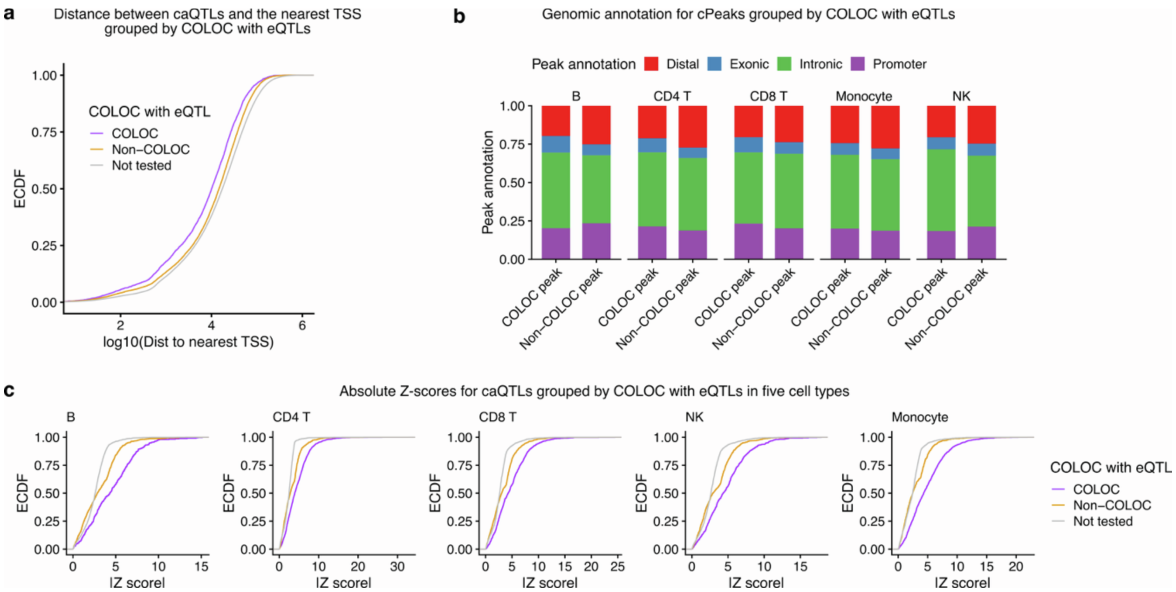

Figure S12. Features of colocalized eQTL and caQTLs, related to Figure 5. a, Cumulative distribution of distance between a caQTL and its nearest TSS, grouped by if the caQTL colocalizes with eQTL or not, or not tested for colocalization. **b**, Genomic annotations for cPeaks that colocalized with eQTLs or not in each cell type. No significant differences are observed. **c**, Cumulative distribution of absolute Z scores of caQTLs that colocalize with eQTL or not, or not tested for colocalization.

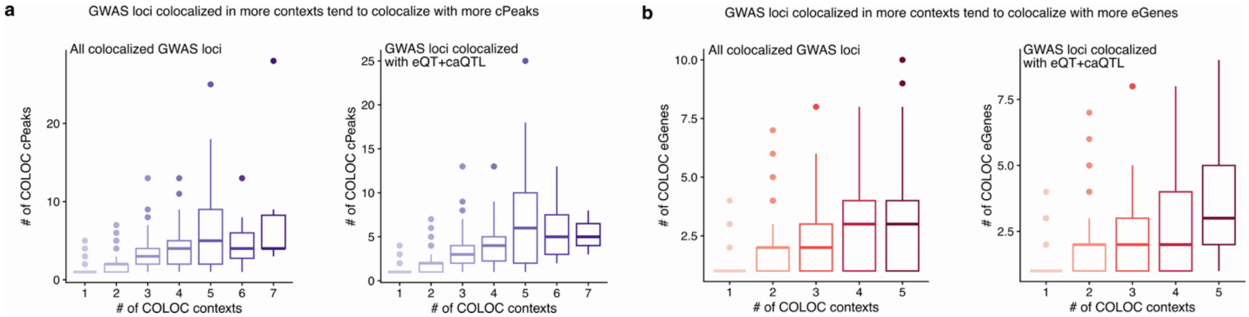

Figure S13. Pleiotropy in caQTL-GWAS and eQTL-GWAS colocalizations, related to Figure 5. a, Boxplot showing the number of colocalized peaks for each GWAS locus as a function of the number of contexts where colocalization is detected. Left, all GWAS loci colocalized with caQTLs. Right, GWAS loci colocalized with both caQTLs and eQTLs. **b,** Boxplot showing the number of colocalized genes in DICE for each GWAS locus as a function of the number of contexts where colocalization is detected. The 15 DICE cell-types and subtypes were mapped to 5 common immune cell-types. Left, all GWAS loci colocalized with caQTLs. Right, GWAS loci colocalized with both caQTLs and eQTLs.

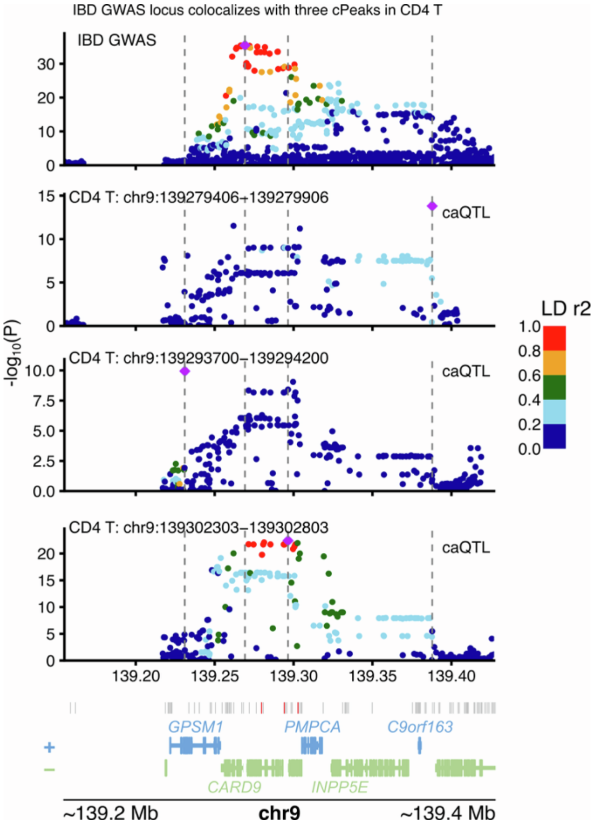

Figure S14. IBD GWAS locus near CARD9 colocalizes with caQTLs in CD4 T cells, related to Figure 5. LocusZoom plots for three cPeaks in CD4 T cells that colocalize with the same IBD GWAS locus near gene *CARD9*.

Supplemental References

1. Benaglio, P., Newsome, J., Han, J.Y., Chiou, J., Aylward, A., Corban, S., Miller, M., Okino, M.-L., Kaur, J., Preissl, S., et al. (2023). Mapping genetic effects on cell type-specific chromatin accessibility and annotating complex immune trait variants using single nucleus ATAC-seq in peripheral blood. PLoS Genet. *19*, e1010759.
